# AMBRA1 and SQSTM1 expression pattern in prostate cancer

**DOI:** 10.1007/s10495-015-1176-3

**Published:** 2015-09-30

**Authors:** Laura Falasca, Francesco Torino, Matteo Marconi, Manuela Costantini, Vincenzo Pompeo, Steno Sentinelli, Laura De Salvo, Mario Patrizio, Cristiano Padula, Michele Gallucci, Mauro Piacentini, Walter Malorni

**Affiliations:** Laboratory of Cell Biology and Electron Microscopy, National Institute for Infectious Diseases I.R.C.C.S. ‘L. Spallanzani’, Rome, Italy; Department of Systems Medicine, Chair of Medical Oncology, Tor Vergata University of Rome, Rome, Italy; Department of Drug Research and Medicine Evaluation, Istituto Superiore di Sanita’, Rome, Italy; Department of Urology, Regina Elena National Cancer Institute, IRCCS, Rome, Italy; Laboratory of Molecular Medicine and Biotechnology, University Campus Bio-Medico of Rome, Rome, Italy; Department of Pathology, Regina Elena National Cancer, Institute, IRCCS, Rome, Italy; Life Line Lab S.R.L., Guidonia, RM Italy; Department of Biology, University of Rome ‘Tor Vergata’, Rome, Italy; Istituto San Raffaele Pisana, Rome, Italy

**Keywords:** Prostate cancer, Autophagy, Immunohistochemistry, Electron microscopy

## Abstract

Prostate cancer is among the most commonly diagnosed male diseases and a leading cause of cancer mortality in men. There is emerging evidence that autophagy plays an important role in malignant cell survival and offers protection from the anti-cancer drugs in prostate cancer cells. AMBRA1 and the autophagic protein sequestosome-1 (SQSTM1; p62) expression were evaluated by immunohistochemistry and western blot on tissue samples from both benign and malignant prostatic lesions. The data reported in this pilot study demonstrated an increased expression of AMBRA1 and SQSTM1, which were also associated with an accumulation of LC3II in prostate cancer but not in benign lesion. In the present study we found that: (i) at variance with benign lesion, prostate cancer cells underwent SQSTM1 accumulation, i.e., clearly displayed a defective autophagic process but, also, (ii) prostate cancer accumulated AMBRA1 and (iii) this increase positively correlated with the Gleason score. These results underscore a possible implication of autophagy in prostate cancer phenotype and of AMBRA1 as possible cancer progression biomarker in this malignancy.

## Introduction

Autophagy is a cellular stress response and a quality control mechanism involved in the lysosomal degradation of cytosolic components in both physiological and pathological conditions [1]. As a general rule, autophagy acts as a pro-survival mechanism, regulating the turnover of long-lived proteins, protein aggregates, intracellular lipid deposits and damaged organelles, as well as intracellular bacteria and viruses [2, 3]. During autophagy, cargo materials are engulfed in double-membraned vesicles called autophagosomes that then fuse with the lysosomes for subsequent digestion and recycling of their content [4, 5].

In cancer, autophagy may play dual opposite roles: by preventing carcinogenesis on one hand or, on the other, conferring an advantage to cancer cells to survive under various stress conditions, including hypoxia, starvation, cytotoxic drugs and radiotherapy [6–8]. During early tumorigenesis, aberrations in oncogenes and/or tumor suppressor genes frequently inhibit or impair the autophagic process. In this condition, the defective autophagic flux can lead to the accumulation of the cargo-binding protein SQSTM1, which has been reported to represent a direct link between autophagy impairment and tumorigenesis [9, 10]. On these bases, some anticancer agents, e.g., the mammalian target of rapamycin (mTOR) inhibitors such as rapamycin and derivatives such as everolimus, have been introduced in the field of cancer treatments. In fact, these agents have been shown to modulate the activity of proteins involved in autophagic process, leading to the concept that its modulation might represent a valuable therapeutic target in patients affected by several malignancies, including prostate cancer [11, 12].

Prostate cancer (PCa) is the second-leading cause of cancer-related mortality after lung cancer in men from developed countries [13]. In its early stages, primary tumor growth is dependent on androgens. Thus, it can generally be controlled by androgen deprivation therapy. However, in some patients, the disease progresses to castration-resistant prostate cancer (CRPC), a lethal form in needed of more effective treatments [14].

The initial management plan for men with newly diagnosed PCa depends upon a pre-treatment assessment of the personal risk of loco-regional recurrence or disseminated disease combined with patient age, life expectancy and overall medical condition. Key factors in this assessment include clinical staging of the extent of disease, the pre-treatment serum prostate specific antigen (PSA) levels, and the Gleason score in the pre-treatment biopsy [15]. Surgery and radiation therapy may cure organ-confined disease, while hormonal agents inhibiting androgen production and/or blocking the function of androgen receptor (AR) are active in the locally advanced/metastatic hormone-sensitive PCa. However, due to the emergence of tumor cell clones resistant to androgen deprivation therapy, newer anti-androgen agents (abiraterone acetate, enzalutamide) or cytotoxic chemotherapy are currently standard treatment options. Moreover, sipuleucel, an anticancer vaccine and radium 231, a radionuclide, may improve survival in CRPC patients with “indolent” disease or bone metastases, respectively [16]. However, although these recent insights provided some useful clues to the physicians, the need for more precise prognostic and predictive factors helping clinicians in better defining prognosis and treatment of patients affected by PCa appears still mandatory.

As concerns the role of autophagy in cancer onset and progression, a possible critical role has recently been underscored by several studies. Proteins involved in autophagy have been evaluated either as molecular targets for cancer treatment or as potential prognostic and/or predictive factors in tumor development. Various autophagy-related proteins are highly expressed in the breast, lung, endometrial, melanoma and urothelial tumours, and are significantly associated with local tumor aggression and worse prognosis [17, 18]. As concerns PCa, autophagy and its alterations have been proposed either as determinants or drug targets [19]. For instance, an impairment due to mono-allelically deletion of BECLIN1, a core protein of autophagy, required for autophagosome formation, has been observed so that this molecule could represent a novel matter of studies in the field of cancer [20, 21].

In this work, the expression of autophagy-related proteins has been analysed in PCa human samples from a homogeneous cohort of patients affected by locally advanced PCa. The pro-autophagic protein AMBRA1, a member of the autophagy signalling network in vertebrates which is involved in autophagosome formation, acting as a positive regulator of BECLIN 1, and the p62 (SQSTM1/sequestosome), that confers selectivity to autophagy by playing a critical role in recognizing/loading cargo into autophagosomes, have been evaluated. The results have been compared with “normal” (peritumoral tissue) and benign prostatic hyperplasia samples, and the correlation with Gleason Score, PSA levels and relapsing disease have been evaluated for each marker considered.

## Materials and methods

### Patients

This is a retrospective study based on formalin-fixed paraffin-embedded archival material obtained from the Regina Elena National Cancer Institute. Specimens of human prostate tissues derived from patients who underwent curative surgical resection for PCa, and pathological examination confirmed the biopsy diagnosis of prostate adenocarcinoma. The study was based on 26 consecutive patients who had not received preoperative treatment. According to the TNM AJCC/UICC staging system, all 26 cases the disease was staged IIIA (T3N0). The selection of cases was performed to include patients who underwent surgery between 2006 and 2008, in order to obtain a follow up period of at least 5 years for each single patient. Postoperatively, the patients were scheduled for regular follow-up visit, and biochemical and clinical progression data were available.

The study also included benign prostate hyperplasia (BPH) samples (12), collected from patients undergoing radical prostatectomy. Paired normal specimens were obtained from an area that was at least 1 cm away from any cancerous tissue and did not contain either cancer cells.

In addition, fresh material from surgical specimens of four patients was collected to perform electron microscopy and western blot analysis.

The study design was approved by the research ethics committee of the Institute and informed consent for performing this study was obtained from all patients.

The following biochemical and pathological parameters were recorded: total PSA, Gleason score, surgical margins infiltration, extraprostatic extension, lymph node metastasis, and TNM staging system (based on the AJCC Cancer Staging Manual, Seventh Edition, 2010, Springer New York, Inc.).

### Immunohistochemical analysis

Formalin-fixed, paraffin-embedded prostate epithelia sections were used. Sections, cut at 4 μm were either stained with Mayer’s hematoxylin and eosin (H&E) for histopathological examination, or used for subsequent immunohistochemical analysis.

For immunohistochemistry, sections, mounted on slides, were deparaffinized in xylene, incubated for 5 min each in 100, 90, 70, and 50 % ethanol for rehydration and immersed in 10 mM sodium citrate, pH 6.0, and microwaved for antigen retrieval. Endogenous peroxidase activity was blocked by 3 % H_2_O_2_ for 5 min. After rinsing in phosphate-saline buffer (PBS) nonspecific antibody binding was reduced by incubating the sections with normal goat serum for 5 min. Sections were washed in PBS/1 % BSA buffer and incubated with primary antibodies: rabbit anti-p62/SQSTM1 from MBL (Woburn, MA, USA) 1:400, and rabbit anti AMBRA1 (ProSci) 1:100 were used. Reactions were visualized using a streptavidin–biotin-immunoperoxidase system with DAB (Biogenex, San Ramon, CA) as chromogen substrates. Negative control staining was performed by omitting the primary antibody. Sections were counterstained in Mayer’s acid hemalum.

### Interpretation and quantification of the staining

The extent of immunoreactivity in the samples was assessed by two authors, using the same microscope by using a ×40 objective with a field diameter of 0.52 mm. Staining intensity was interpreted and scored on a semi-quantitative subjective scale as follows: none, (+) weak, (++) moderate, and (+++) strong. Immunohistochemical results were evaluated considering the overall proportion of positive cells: no staining; 1 for 1–50 % of cells with positive staining, 2 for >50 % of cells with positive staining.

In addition, for each case the intensity and frequency of label were then added to produce a semi-quantitative final score. This scoring system takes into consideration the proportion of positive cells (scored on a scale of 0–2) and staining intensity (scored on a scale of 0–3). The proportions of positive cells and intensity were then added to produce final scores of 0 or 2–5.

The slides were independently scored by 2 clinical pathologists in a double-blinded manner.

### Western blot analysis

Tissue samples were lysed in lysis buffer containing 1 % Triton X-100, 10 mM Tris–HCl (pH 7.5), 150 mM NaCl, 5 mM EDTA, 1 mM Na_3_VO_4_ and 75 U of aprotinin and allowed to stand for 20 min at 4 °C. The tissue suspension was mechanically disrupted by Dounce homogenization (ten strokes). The lysate was centrifuged for 5 min at 1300×*g* to remove nuclei and large cellular debris. After evaluation of the protein concentration by Bradford dye reagent assay (Bio-Rad, 500-0006), the lysate was subjected to 8 % (for AMBRA1) or 15 % (for other antigens) sodium-dodecyl sulphate polyacrylamide gel electrophoresis (SDS-PAGE). The proteins were electrophoretically transferred onto polyvinylidene difluoride (PVDF) membranes (Bio-Rad, 162-0177). Membranes were blocked with 5 % defatted dried milk in TBS, containing 0.05 % Tween-20 and probed with rabbit polyclonal anti-LC3 antibody (MBL Int Corporation, PD014), with rabbit polyclonal anti-AMBRA1 (NOVUS, NBP1-07124), with rabbit polyclonal anti-p62 SQSTM1/sequestosome antibody (Sigma, P0067) or with anti-alpha-tubulin Mab (Sigma, T6199) Bound antibodies were visualized with horseradish peroxidase (HRP)-conjugated anti-rabbit IgG (Sigma, A1949) or anti-mouse IgG (Sigma, A9044) and immunoreactivity assessed by chemiluminescence reaction, using the ECL Western detection system (Amersham, RPN2106).

### RNA isolation and quantification

Total RNA was extracted from the three representative patients by using SV total RNA isolation system (Promega, Madison, USA). cDNA out of total RNA was synthesized by using the high capacity cDNA archive kit (Applied Biosystems, Foster City, CA, USA). mRNA expression levels for AMBRA1 and SQSTM1 were quantified with real-time TaqMan RT-PCR using 7500 real-time PCR system (Applied Biosystems, Foster City, CA, USA). TaqMan reactions were carried out in 96 well plates using cDNA, TaqMan universal PCR mastermix, pre-optimized and pre-formulated TaqMan^®^ gene expressions assays including specific primers and fluorescent probes for humans, and water to a final volume of 50 μL according to manufacturer’s instructions. The codes for each gene expressions assay were derived from the online applied biosystems catalogue for quantitative gene expression analysis. Glyceraldehyde 3-phosphate dehydrogenase (GAPDH) was used as an endogenous control. No reverse transcriptase and no template controls were used to monitor for any contaminating amplification.

### Electron microscopy

Tissue samples were fixed with 2.5 % glutaraldehyde (Assing Spa, R1012) in 0.1 M cacodylate buffer for 1 h at 4 °C (sodium cacodylate trihydrate, Sigma-Aldrich, C4945), and postfixed in 1 % osmium tetroxide (Sigma-Aldrich, 75632) in 0.1 M cacodylate buffer for 1 h. Samples were then dehydrated in graded ethanol and embedded in Epon resin (AGAR 100, Agar Scientific R1045). Ultrathin sections were stained with 2 % uranyl acetate (Sigma-Aldrich, 73943) and observed under a Zeiss EM900 transmission electron microscope. Images were captured digitally with a Mega View II digital camera (SIS; Zeiss).

For morphometric analysis, the number of vesicles per cell was counted under transmission electron microscope at the same magnification (×12,000, 48 μm^2^). Electron micrographs obtained (at least 50 images/sample) were examined and values were expressed as mean ± SEM per field.

### Statistical analysis

Expression rates of proteins analysed were calculated as the proportion of positive reactivities within total cells per high-power field. Statistical analysis was carried out using the Student’s *t* test correlation test. The *χ*^2^ test was used to test for a potential association between study variables of interest. Differences were considered significant when *P* values were <0.05.

## Results

### Patients

In this study the expression of autophagic markers has been evaluated in human prostate tissues obtained from patients who had not received hormone therapy, including normal adjacent tissues from PCa patients (*n* = 26), and BPH (*n* = 12).

The clinical and pathological features of all patients enrolled in this study are reported in detail in the Table 1. The mean age of the patients at the time of surgery was 63 years (range 52–76 years), with an average serum PSA level of 12.3.

**Table 1:** Clinical and pathological characteristics of patients

Archived material	
*Histopathology* (*slides*)	
Number of patients	26
Age years (mean)	52–76 (63)
PSA at diagnosis mean (range)	12.3 (4.8–33)
Stage	III pT3a
Gleason score	
6 (3 + 3)	5
7 (3 + 4)	5
7 (4 + 3)	12
8 (4 + 4)	4
Perineural invasion	17
Follow-up data (5 years)	
Overall mortality	3
Death, due to prostate cancer	1
PSA relapse	4
Local recurrence	7
Metastasis	2
Any tumor excluding PCa	2
Benign hyperplastic prostate samples	12
*Fresh specimens*	
Number of patients	4
Age years (mean)	63–74 (69)
Stage III	
pT3a, N0, M0	1
pT3b, N0, M0	2
Stage IV	
pT3, N1, M0	1
Gleason score	
7 (3 + 4)	1
7 (4 + 3)	2
8 (4 + 4)	1

### Immunohistochemical analysis

The subcellular localization and expression of AMBRA1 and SQSTM1 proteins were observed and scored by IHC on large section of prostate adenocarcinoma from 26 patients.

**Fig. 1:**
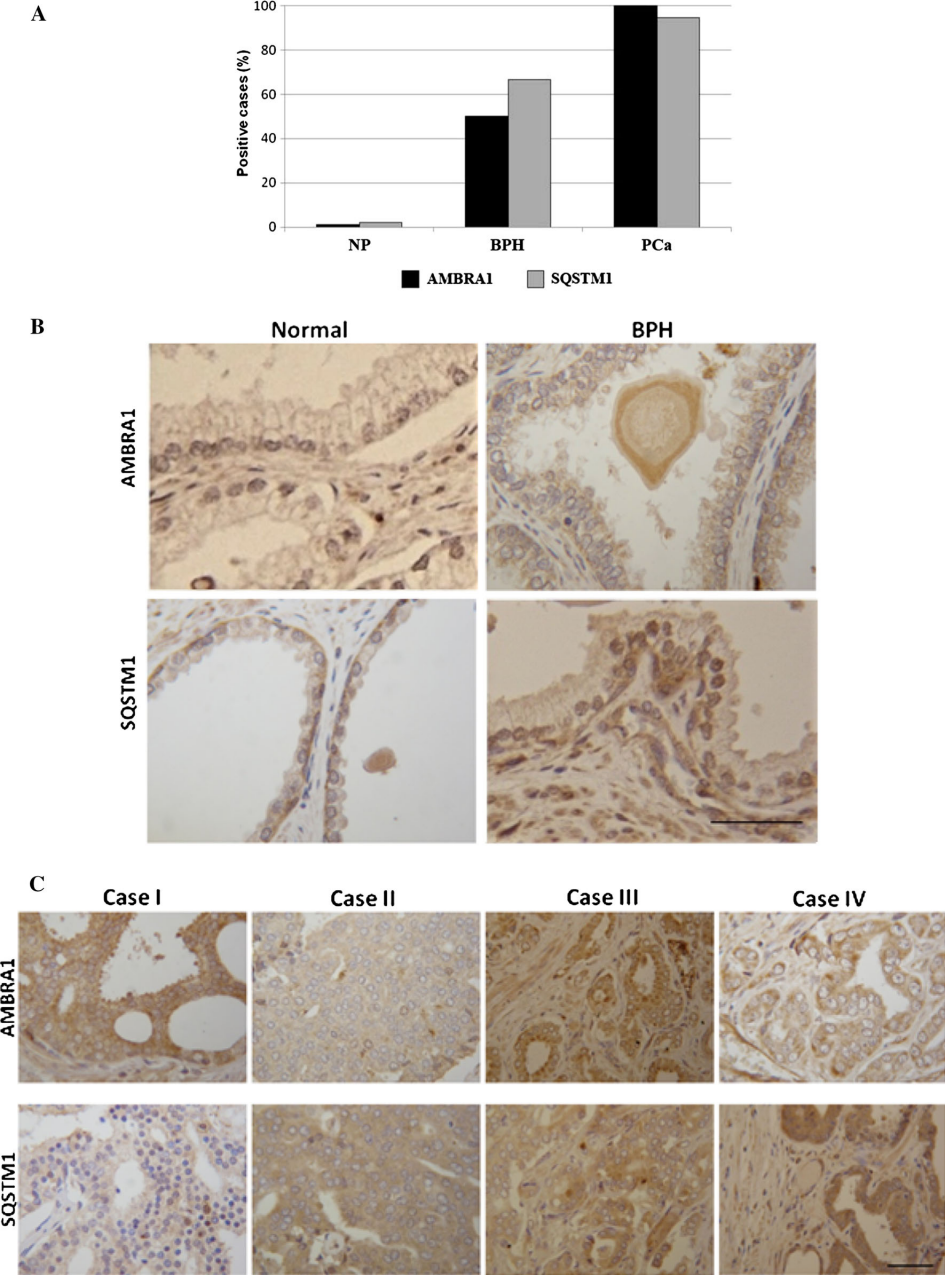
**a** Percentage distribution of AMBRA1 and SQSTM1 expression in normal prostate (NP), benign hyperplasia (BPH) or neoplastic prostate (PCa). Protein expression in BH and PCa lesions was analyzed by *χ* ^2^ test. A statistically significant association was found between both proteins overexpression and PCa lesions: AMBRA1 (*P* < 0.001; CI 95 %); SQSTM1 (*P* < 0.05; CI 95 %). **b** Representative immunohistochemistry micrographs showing AMBRA1 and SQSTM1 staining of normal and human benign prostatic hyperplasia (BPH). Normal prostate glands, with tall columnar epithelial lining cells, display negative staining for both proteins. BPH photomicrographs showed a positive labeling of AMBRA1 and SQSTM1, the staining intensity is always weak. SQSTM1 expression was also found in stromal cells and sometimes in the nucleus of luminal epithelial cells. *Bar* 50 μm. **c** Immunohistochemical expression of AMBRA1 and SQSTM1 in PCa specimens. Representative images of two contiguous sections of four samples (cases I–IV) from patients diagnosed with same Gleason stage are showed. Both proteins exhibit heterogeneous intensity of staining. *Bar* 50 μm

The immune reactivity of AMBRA1 and SQSTM1 was localized in the cytoplasm (and/or nucleus) of epithelial cells of PCa and BPH tissues, while no signals (or at least very faint) were detected in the normal prostatic tissues (Fig. 1a, b). The benign prostatic hyperplastic regions showed a positive staining for AMBRA1 in 6/12 cases (50 %) (Table 2; Fig. 1a). The immunoreactivity was found in a proportion of cells ranging from <10 up to 50 % (Table 2), usually with a weak intensity (Fig. 1b). In the tumor samples, AMBRA1 expression was instead observed in 100 % of cases (Fig. 1a). The staining was observed in a high proportion of cells >50 in 58 % of cases (Table 2).

**Table 2:** Expression of AMBRA1 and SQSTM1 in hyperplastic and neoplastic prostate tissue

	Positive cases	Proportion of positive cells (%)		
		<10	10–50	>50
Hyperplasia (*n* = 12)				
AMBRA1	6/12	46.2	53.8	
SQSTM1	8/12	34.4	65.6	
PCa (*n* = 26)				
AMBRA1	26/26		42.0	58.0
SQSTM1	24/26	5.3	31.6	63.1

In benign prostate hyperplasia SQSTM1 positive staining was observed in 8/12 cases (66.7 %) of cases (Table 2; Fig. 1a); the proportion of the cells stained was between 10 and 50 %, usually with a weak intensity (Table 2; Fig. 1b). In the neoplastic tissue the expression of SQSTM1 was found in 92.3 % of cases (Fig. 1a). Staining was present in a proportion of cells >50 in 63 % of cases (Table 2).

To note a considerable variability was detectable in the expression of AMBRA1 and SQSTM1 in the neoplastic prostate. The staining was heterogeneous (Fig. 1c), even though the vast majority of cases displayed a moderate intensity of staining (68.5 % for AMBRA1 and 57.9 % for SQSTM1) (Table 3).

**Table 3:** Intensity of AMBRA1 and SQSTM1 expression in prostate tissue

	BPH (*n* = 12)	PCa (*n* = 26)
AMBRA1		
Negative	6	0
Weak (+)	6	3
Moderate (++)	0	18
Strong (+++)	0	5
SQSTM1		
Negative	4	1
Weak (+)	5	3
Moderate (++)	3	15
Strong (+++)	0	7

*BPH* Benign prostate hyperplasia, *PCa* prostate cancer

**Fig. 2:**
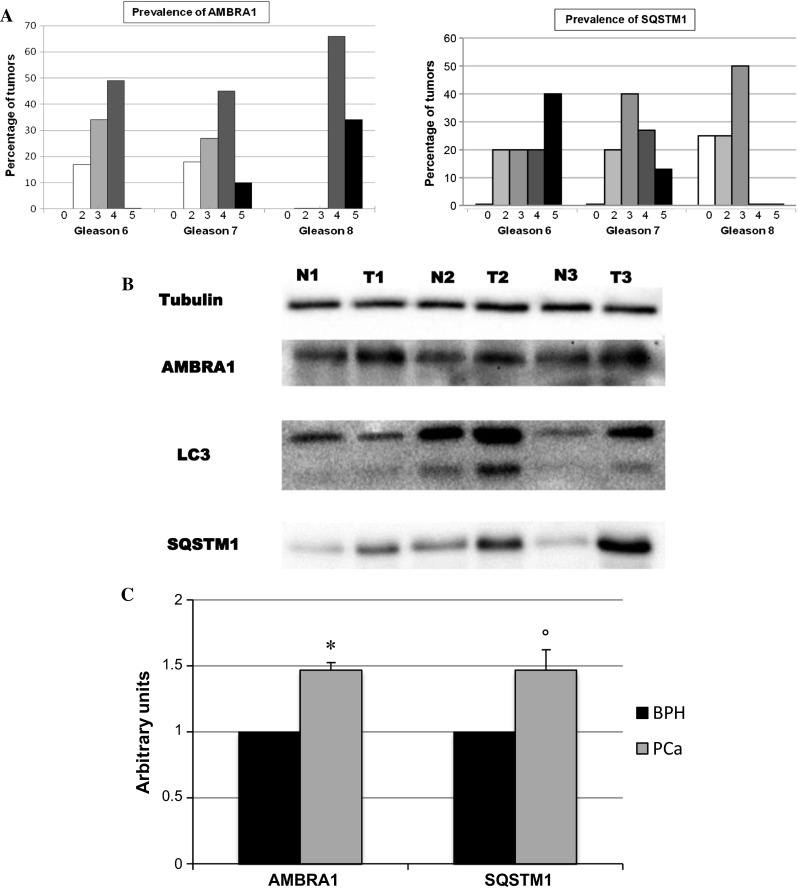
**a** Immunohistochemical results showing the prevalence of AMBRA1 (*left*) and SQSTM1 (*right*) based on a semi-quantitative total score. Frequency distribution of the protein is reported according to the Gleason grade classification (GL6–GL8). A trend of AMBRA1 high score values correlation with the higher grade of Gleason score is visible. As opposite the levels of SQSTM1 positivity appeared higher in tumors with a lower Gleason score (Gleason 6). **b** The protein levels of AMBRA1, LC3 and SQSTM1 were analyzed by western blot from fresh tissue samples. Tubulin was also detected as the control of sample loading. Representative western blots were shown. *N* normal tissue, *T* PCa tissue. **c** mRNA expression of AMBRA1 and SQSTM1 clearly shows that a significant upregulation of these genes occurs in PCa in comparison with BPH. * *P* < 0.01; ° *P* < 0.05

Analyzing the levels of AMBRA1 expression in PCa and BPH prostate tissues, we found significant differences: in 15 out of 26 PCa cases (58 %), the percentage of AMBRA1 positive cells was very high (more than 50 %) whereas all the positive BPH tissues (6 out of 12) displayed a lower positivity for AMBRA1, i.e., below 50 % (*P* < 0.001) (Table 4). The prevalence of AMBRA1 in PCa was obtained by rescoring the immunohistochemical results to obtain a semi-quantitative final score. This scoring system indicates that levels of AMBRA1 were greater (score 4–5) in tumors with a higher prostate cancer grading system (Fig. 2a, left panel), again suggesting a positive correlation between AMBRA1 positivity and PCa lesions. Concerning the prevalence of SQSTM1, no correlation was found between the positivity of the protein and the grade of PCa lesions; the levels of SQSTM1 appeared higher in tumors with a lower Gleason score (Gleason 6), with respect to higher Gleason scores (GL7 and GL8) (Fig. 2a, right panel).

**Table 4:** Comparison of AMBRA1 immunostaining between BPH and PCa

AMBRA1 expression	BHP	PCa	Total
≤50 %	12	11	23
≥50 %	0*	15*	15
	12	26	38

* *P* < 0.001

### Quantitative analysis of the levels of autophagic markers

In order to define whether the observed accumulation of AMBRA1 and SQSTM1 reported above were due to a block of autophagic flux in PCa, we analyzed by immune blotting the processing of LC3-I protein that is modified into the PE-conjugated form, LC3-II. This isoform is membrane-associated and is a typical hallmark of autophagosome formation. LC3-II is located in the inner autophagosomal membrane and digested upon fusion with lysosomes [22], thus the accumulation of LC3-II might suggest that the autophagic flux is down-regulated. As showed in Fig. 2b, an increase of LC3-II was found in tumor samples with respect to controls. Densitometric evaluation, after normalization with tubulin, revealed a mean value of 88 % increase of LC3-II protein level in tumoral tissues even though, as conceivable, a marked variability was observed among patients analyzed (data not shown).

Similar results were obtained when the levels of AMBRA1 were analyzed (Fig. 2b). A mean increase of 40 % was found in PCa, compared with controls. Analysis of SQSTM1 revealed a strong accumulation of the protein in tumors (Fig. 2b) a fourfold increase (densitometric mean value; data not shown), which is indicative of an impairment of autophagic degradation. These data clearly suggest that in prostatic cancers an impairment of the autophagic flux could occur.

These data were confirmed by checking mRNA gene expression levels of AMBRA1 and SQSTM1 in three representative patients. As shown in Fig. 2c, the expression levels of these genes were found significantly higher in samples from PCa in comparison with BPH.

### Electron microscopy

**Fig. 3:**
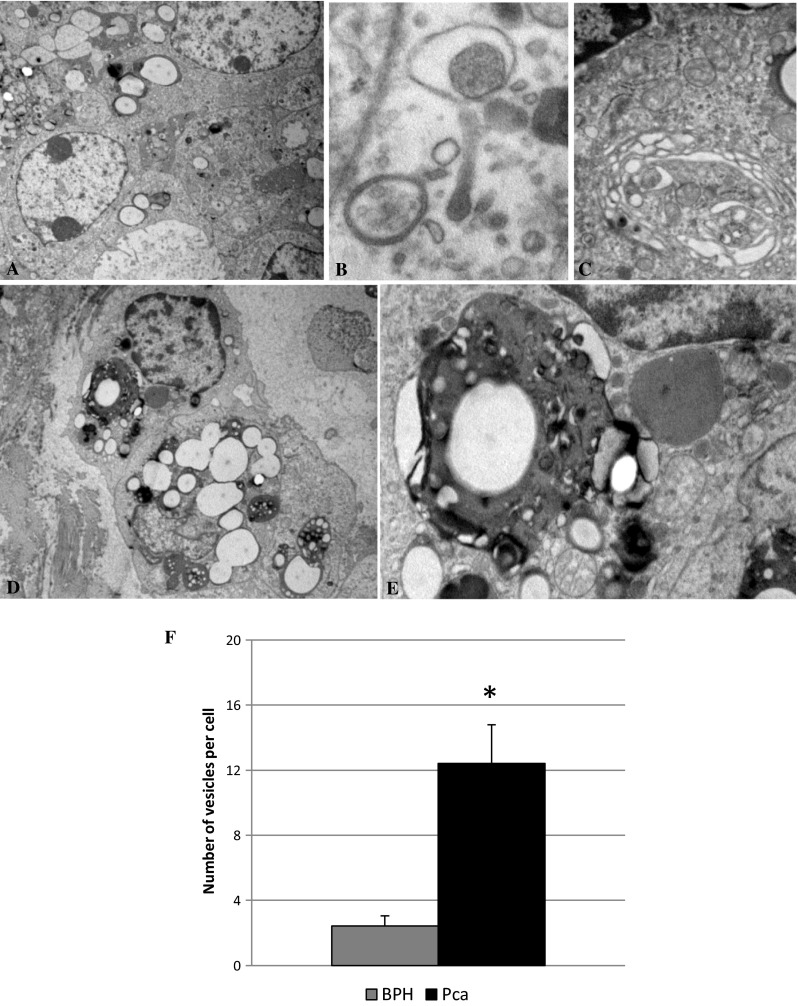
**a** Electron microscopy images show different morphology exhibited by cells in prostate cancer tissue. The presence of autophagic vacuoles (**b**) and multilamellar bodies (**c**) can be observed in some cells; extensive vacuolization (**d**) and pleomorphic bodies containing lipid droplets and bits of membranes (**e**), were found in different cells. **f** Quantitative analysis of vacuoles in PCa and BPH cells. Note that the number of vacuoles is significantly higher in cells from PCa in comparison with those from BPH * *P* < 0.01

In order to get further insights into autophagy levels in the setting of PCa we employed electron microscopy to analyze tissues at the ultrastructural level. Phenotypical characteristics of PCa cells are often heterogeneous; they can display an extremely dedifferentiated morphology, containing only a few organelles, as well as a well-differentiated morphology with the usual balance of the various intracellular components in the same lesion (Fig. 3a). Furthermore, this variety of phenotypical features of cancer cells also reflected the heterogeneous levels of autophagic activity. In fact, in prostate cells, the electron microscopy analysis revealed the presence of autophagic vacuoles (Fig. 3b) and multilamellar bodies (Fig. 3c). An extensive vacuolation and the presence of large vesicles containing lipid droplets and membranous material, that is usually indicative of an impairment of autophagolysosomes maturation and a block in their content degradation (Fig. 3d, e), could often be observed. We performed counts of vacuoles on a series of electron micrograph images from BPH and PCa samples. We found that vesicle accumulation was significantly higher in cells from PCa samples than in cells from BPH samples (Fig. 3f). These data further suggest that in the PCa cells an impairment of autophagic flux leading to remnants accumulation, i.e., a defective autophagy, could take place.

## Discussion

Despite intensive investigation, the significance of autophagy in the progression of human tumors remains poorly understood. Considering that autophagy plays a critical role at the interface of cell survival and cell death, the understanding of the underlying signaling pathways that regulate its impact on cell death versus survival decisions is critical for the further exploitation of autophagy as a strategy for cancer therapy. Anyway, autophagy has been associated either with pathogenetic mechanisms of the disease or with therapeutic intervention. The upregulated expression of Atg5, an E3 ubiquitin ligase which is necessary for autophagosome elongation, has been suggested to play a role in the tumorigenesis of PCa [23]. Conversely, it has been hypothesized that autophagy inhibition may be useful to make PCa cells more sensitive to pro-apoptotic stimuli. Indeed, it has been shown that an autophagy blockade in vitro sensitizes prostate cancer cells toward Src tyrosine kinase inhibitors [24]. These inhibitors can impede androgen-independent growth of prostate cancer cells but do not induce significant apoptosis. In this context, autophagy blockade significantly potentiates pro-apoptotic effects of tyrosine kinase inhibitors [25]. In general, the protective function of autophagy in cancer cells subjected to chemotherapy or radiation generated an intense interest in evaluating autophagy inhibition as a possible clinical strategy to counteract therapeutic resistance in PCa [26]. In androgen-independent prostate cancer cells, it has been also shown that autophagy induction may sensitize cells to apoptotic stimuli [27, 28] and radiation [29]. These data paradoxically suggest that, depending on the cellular features, either the induction or the inhibition of autophagy might provide therapeutic benefits to PCa patients.

The aim of this study was to assess if some proteins involved in the modulation of the autophagic process in PCa tissue samples could be related with Gleason histologic scores, clinical stages, and serum PSA levels. In particular, we have focused our attention on the regulation of autophagy at two levels by analyzing two pro-autophagic proteins, AMBRA1 and SQSTM1, which play a key role in the omegasome formation and the cargo recruitment, respectively. So far, most of the studies have been performed using prostate cells in culture or analyzing the effect of chemotherapeutic agents. By using different experimental approaches we found that both proteins are overexpressed in PCa in vivo. These findings were also confirmed by the accumulation of LC3II, thus suggesting that autophagy takes place but, also, that, as suggested by SQSTM1 cargo protein accumulation, it could be rather impaired. Accordingly, in the case of AMBRA1 we detected a good correlation with the Gleason score, whereas the SQSTM1 expression levels, previously suggested as not altered in PCa [30], seem to not correlate with prostate cancer grading system. This could imply that cancers with a high AMBRA1 expression could be more aggressive and have a worse prognosis. Furthermore, results dealing with mRNA of AMBRA1 and SQSTM1 indicated that these genes, significantly upregulated in samples from PCa in comparison with BPH, could contribute to PCa progression. Increased levels of SQSTM1 seem to suggest that, as mentioned above, the autophagic process in PCa cells, although potent, could be defective, leading to the accumulation of “not-digested” SQSTM1. However, since literature dealing with this matter appears still at the beginning [31, 32], further studies on the implication of autophagy in PCa progression appear as mandatory.

Tumor heterogeneity is a critical issue and a major limitation for molecular diagnostics and targeted cancer therapy. Diagnostic accuracy of a molecular assay may be limited if the analyzed biomarker is only present in a fraction of a tumor. Multifocality is a well-known feature of PCa and is found in from 60 to 90 % of these tumors [33]. This surely represents a critical factor in this type of cancer so that the search for novel and more appropriate biomarkers is underway in several laboratories. Importantly, a significant breach is now evident among the huge amount of studies on the genetic characterization of PCa, which has limited translation to clinical practice. From a clinical point of view, this balance should be urgently shifted towards translation. Nevertheless, strict control of the significance of the new markers is necessary against the common clinical and pathological variables (e.g., Gleason score). We propose that further search on the prognostic impact of AMBRA1 on prostate cancer could provide useful information for a more tailored and predictive evaluation of this heterogeneous form of cancer
